# Annotations

**Published:** 1895-01-12

**Authors:** 


### ANNOTATIONS.

### Bacteria and their Products.

IT will help the elementary student of chemistry, and every doctor may claim to be so much of a chemist as an elementary student, to be told, as the members of the Epidemiological Society were told by Dr. Klein at their last meeting, that all the forms of bacteria produce an action upon the organic fluids in which they grow which is strictly analogous to that produced by the yeast fungus on saccharine solutions. Just as the action of yeast in saccharine solutions always results in the evolution of the same definite products, so all bacteria, in known media, produce bodies of definite chemical composition. Of this there is no doubt whatever. From the point of view of Pathogeny, the most important of these definite products are the ptomaines, or albumoses, which are developed in albuminous fluids. Pathogenic bacteria are probably never pathogenic by their mere presence, or even by their almost infinite multiplication, but by the toxins, ptomaines, or albumoses, which they produce in the course of their life history. The toxins can be separated, as they were by Roux and Yersin in the case of the diphtheria bacillus; and they are then found capable of inducing all the ordinary effects of the disease except one, but that a most important one. They do not produce a disease which is communicable by ordinary infection. It is clear, then, that the bacillus, as well as its products,' has a fundamental part to play in the evolution of specific diseases, and in this connection we are still permitted to indulge the hope that the doctrine of phagocytosis may yet come out scatheless from the fires of laboratory and clinical criticism.

### De Motu Cordis et Sanguinis.

"*Sed solam veritatem sector, et omnem tum operam, tum oleu eo contuli, ut aliquid bonis gratum, doctis commodum, et rei litterariae utile in medium proferre possim.*" Thus William Harvey in his dedication to Dr. Argent, president of the Royal College of Physicians, of his great work, "De Motu Cordis," in the year 1628. The Latin may be thus rendered: "Of truth alone I am a follower; and I can say from my heart that I have studied to the utmost limit of my power to produce something which shall be pleasing to the virtuous, profitable for the learned, and of lasting service to the world of letters." A new edition of Dr. Harvey's great scientific work has been published by Mr. George Moreton, of Canterbury, the enterprising publisher who last year issued to more than a thousand subscribers Sir Thomas Browne's classical work, "Religio Medici." Our object here is not to review, or even to give a customary notice of the reissue of a venerable publication, but to call attention to the last clauses of the penultimate sentence of Dr. Harvey's modest and learned dedication. He desired, he tells the College of Phys'cians, "to produce a work which should give pleasure to the virtuous and be of lasting service to the world of letters as well as convenient and useful to the learned practitioners of the medical art." In other words, he hoped and believed that he was not merely writing a "craftsman's treatise," but bringing out a permanent contribution to literature. It is here that the cultured ancients of our profession were so far in advance of their modern fellows. The doctor who writes a book to-day, however large and wide his subject may be, seldom thinks of his work as a contribution to literature. But he should. Not so thinking, he degrades his subject; and he has his reward. His book seldom becomes "permanent" in any form, either as literature or as a manual of craftsmanship. Those who cannot turn medicine into literature should seldom write. Now and again they may produce brief papers on special occasions, but authorship they should rigidly eschew. If they would but put a self-denying ordinance upon themselves in this regard they would render a great service to their own reputations, and a still greater to the fair fame of medicine.

### The Prison as a Reformatory.

ONE advantage of comparatively long sentences, in cases of crimes obviously arising from habitual drunkenness, is that it is only by such sentences that any opportunity is given for reform. A week in jail may be punitive, but it certainly is no help or assistance on the path of reformation. The time is too short to break the craving, and the *régime* tends even to aggravate it. To a person accustomed to alcoholic stimulants, few things could tend more to the production of that flabby sense of emptiness and weak vitality, which experience teaches him is best relieved by something both hot and strong, than the hard bed and low diet inflicted on the short time prisoner. His first craving on discharge is for a "nip"; a friend who will then stand a drink is a friend indeed; in truth, such a friend is not often lacking, and thus the whole miserable game is played over again. It is only by a sentence long enough, not only to break the habit, but long enough also to feed up the dyspeptic and debilitated frame, and so remove the miserable "sinking" and sense of want from which these people suffer, that any reformatory effect can be produced. But that such an effect can be produced, and that our prisons judiciously, sa well as judicially, used may be made truly reformatory institutions, there can be no doubt. This was recently amusingly illustrated at a banquet given by Alderman Sir Stuart Knill and the members of the City Police Committee to the Lord Mayor and the Sheriffs and the metropolitan police magistrates, when Sir John Bridge evoked much laughter and applause by quoting from a letter he had recently received from a man who thanked him sincerely for having given his wife six months' imprisonment, which had changed her, he said, from being a drunken scold to a steady, sober woman, with whom, as in their early married life, it was a pleasure to dwell. This is, doubtless, by no means a unique experience, and there can be little question of the frequent efficacy of abstinence in the cure of the habit of drinking. To remove the craving, however, another habit must be substituted, viz., that of taking food, and this is a view of the matter worthy of the consideration of all prison officials.

